# Photodynamic Therapy Combined with Gemcitabine for Prostate Cancer Using Nanoprobe for in-vivo CEST Imaging

**DOI:** 10.5812/ijpr-169821

**Published:** 2026-04-28

**Authors:** Qibo Fu, Qian Liu, Xianyou Zeng, Laoji Wang, Jierou Yan, Ding He, Jilin Xie

**Affiliations:** 1 Department of Urology, Jiading District Central Hospital Affiliated Shanghai, University of Medicine & Health Sciences, Shanghai, China; 2 Department of Operating Room, Huai'an Clinical Medical College of Jiangsu University (Huai'an Hospital of Huai'an City), Huai'an, China; 3 Department of Urology, The Affiliated Hospital of Jinggangshan University, Ji’an, China; 4 Department of Oncology, The First Affiliated Hospital of Anhui Medical University, Hefei, China

**Keywords:** Chemical Exchange Saturation Transfer Imaging, Pyroptosis of Cells, Nanoprobe, Prostate Cancer

## Abstract

**Background:**

Current clinical treatment is confronted with challenges such as low drug delivery efficiency, lagging efficacy evaluation, and a high tumor recurrence rate. However, chemical exchange saturation transfer (CEST) imaging, with its molecular specificity and non-invasive monitoring advantages, provides a new path for real-time tracking of the metabolokinetics of nanoprobes.

**Objectives:**

Drug-loaded nanoprobes that self-assembled and had CEST activation imaging capabilities were created, and there in vivo and in vitro CEST activation imaging efficacy and the value of photodynamic sensitization for pyroptosis treatment of prostate cancer cells were evaluated.

**Methods:**

A self-assembly strategy was adopted to construct a nanoprobe (GC) coloaded with gemcitabine (Gem) and the photosensitizer chlorin e6 (Ce6), and a scanning electron microscope was used. Scanning electron microscope (SEM), dynamic light scattering (DLS), etc., were used to characterize the in vitro CEST activation imaging efficacy of the nanoprobe, and the pH, concentration-, and time-dependent drug release were observed. GC was combined with 2',7'-dichlorodihydrogen fluorescein diacetate ester (2',7'-dichlorodihydrofluorescein diacetate) after laser treatment of prostate cancer cells in mice. The generation of reactive oxygen species (ROS) was detected with a DCFH-DA probe. By using ELISA, the amounts of inflammatory factors such as interleukin-1β (IL-1β) and IL-18 were determined, and calreticulin was detected via immunofluorescence. CRT and high mobility group box 1 protein (HMGB1) were used to evaluate pyroptosis-mediated immunogenic cell death (ICD) effects. A mouse prostate cancer tumor model was constructed to observe the CEST-specific activation effect of the GC nanoprobes in vivo, and its tumor volume was measured, together with the detection of inflammatory substances and ICD markers, in order to assess its pyroptosis-based anticancer activity.

**Results:**

Scanning electron microscope and DLS analyses revealed that the GC nanoprobes had a uniform spherical-like structure. The release of the drug Gem by this drug-loaded nanoprobe under acidic conditions at pH 5.0 was as high as 80%, which was significantly greater than that at pH 7.4 (P = 0.003). DCFH-DA fluorescence staining indicated that photosensitizing pyroptosis mediated by nanoprobes could generate a large amount of ROS. The detection of pyroptosis-related factors revealed that the levels of IL-1β and IL-18 significantly increased (all P < 0.05), whereas the fluorescence of the ICD marker CRT increased and that of HMGB1 decreased. The results of in vivo experiments indicated that the CEST signal at the tumor site was significantly enhanced and reached its peak 4 hours after tail vein injection of the GC nanoprobes. In addition, the results of antitumor treatment in vivo revealed that, compared with those in the control group treated with PBS, the levels of the inflammatory factors IL-1β and IL-18 in the experimental group treated with GC combined with laser irradiation increased, the ICD markers HMGB1 and CRT significantly changed, and the tumors were significantly inhibited (all P < 0.05). The CEST imaging of GC nanoprobes demonstrates excellent diagnostic accuracy in the detection of prostate cancer. The sensitivity reaches 92.3%, the specificity is 88.7%, and the area under the curve (AUC) is 0.95, which is significantly superior to traditional imaging methods. The specific enhancement of CEST signals at the tumor site reaches its peak 4 hours after injection, which is highly consistent with the histopathological results, confirming its clinical value in the early diagnosis and precise treatment guidance of prostate cancer.

**Conclusions:**

The GC nanoprobes successfully prepared in this study can specifically activate CEST imaging, guide the photodynamic sensitization of prostate cancer tumor cells to pyroptosis, and promote the precise ablation of prostate cancer.

## 1. Background

Prostate cancer is one of the malignant tumors with the highest incidence rates among men worldwide. Owing to the lack of therapeutic targets and the susceptibility to metastasis and recurrence, it has become a clinical difficulty, and new therapeutic strategies are urgently needed (1). Pyroptosis is a type of programmed cell death that is jointly mediated by gasdermin (GSDM) family proteins and caspases through the release of damage-associated molecules. DAMPs activate antitumor immune responses and have great potential in the treatment of prostate cancer (2, 3). Conventional chemotherapy drugs (such as cisplatin and doxorubicin) can trigger GSDME (gasdermin-E)/caspase-3-mediated pyroptosis. However, owing to their limitations, such as their fast clearance speed in the blood circulation, they have difficulty accumulating effectively at the tumor site. Therefore, these drugs often fail to achieve the ideal tumor pyroptosis effect (4). Owing to their enhanced permeability and petention (EPR) effects, self-assembled drug-loaded nanoprobes can effectively accumulate at tumor sites. Moreover, they can control the surface properties of nanoparticles through intermolecular interactions. This endows tumors with the characteristics of tumor microenvironment-responsive drug release and has advantages in the regulation of tumor pyroptosis (5). However, pyroptosis induced by a single nanomedicine alone is insufficient to completely kill tumor cells and is prone to causing tumor recurrence and metastasis (6). Relevant studies (7) have shown that the use of photosensitizing pyroptosis can cascade and amplify antitumor immunity and reduce immunosuppression. For example, photodynamic therapy (PDT) can be used as a pyroptosis sensitization method. The tumor might be totally eradicated by producing a significant quantity of lethal reactive oxygen species (ROS) at the tumor site, which would increase the immunogenic cell death (ICD) effect and pyroptosis effect.

Noninvasive visualization of the pyroptosis process in tumor cells is crucial for the precise treatment of prostate cancer and the formulation of personalized treatment plans (8). Traditional pyroptosis detection methods rely on invasive tumor sample testing, whereas noninvasive monitoring of tumor pyroptosis at the in vivo level has rarely been reported. The development of molecular imaging technology provides a new opportunity for the integrated diagnosis and treatment of prostate cancer. Chemical exchange saturation transfer (CEST) imaging, an emerging magnetic resonance molecular imaging technique, can reflect the metabolic information of the tissue microenvironment through changes in exchangeable proton signals. It has great potential for studying pyroptosis in prostate cancer, monitoring drug release, evaluating drug efficacy, etc. (9, 10). Endogenous CEST contrast agents can detect changes in the concentrations of proteins and peptides in tumor tissues and clearly display tumor boundaries, but their diagnostic sensitivity is relatively low (11). In recent years, the introduction of diamagnetic exogenous contrast agents has provided a new technique for the early and precise diagnosis of tumors and the dynamic monitoring of molecular-level changes during the treatment process. Diamagnetic compounds do not rely on the labeling of metal ions. Good CEST imaging contrast can be produced by the chemical exchange between exchangeable protons on the molecular structure (such as -OH, -NH- and -NH2) and the surrounding water molecules. This type of compound has extremely high biocompatibility, and its ability to be easily chemically modified further broadens the imaging function of contrast agent probes (12). As a diamagnetic compound, gemcitabine (Gem), a cytosine nucleoside pyroptosis-inducing drug, contains exchangeable protons such as hydroxyl groups and amides. It has a wide CEST signal resonance at approximately 2 ppm and can provide obvious contrast information in CEST imaging (13-15). Therefore, diamagnetic chemotherapy drugs such as Gem can achieve tumor label-free imaging through CEST imaging technology without the need for exogenous markers. In this study, a self-assembled nanoprobe (GC) coloaded with Gem and the photosensitizer chlorin e6 (Ce6) was designed to induce pyroptosis-based antitumor immune responses. In addition, this nanoprobe can specifically activate CEST imaging in the acidic microenvironment of tumors, which is expected to achieve noninvasive in vivo monitoring of drug delivery and pyroptosis-mediated antitumor efficacy evaluation, thereby providing a new strategy for the precise diagnosis and treatment of prostate cancer.

## 2. Methods

### 2.1. Experimental Animals and Cells

Spf-grade female BALB/c mice aged 4 - 6 weeks were acquired from Shanghai Jihui Experimental Animal Breeding Co., Ltd. The production license number of the experimental animals is SCXK (Shanghai) 2022–0009, and the usage license number of the experimental animals is SYXK (Shanghai) 2024–0030. The mice were allowed to eat freely at intervals of 12 hours, alternating between light and dark, with a temperature of 25 ± 2°C. The mouse prostate cancer PNEC30 cell line was purchased from the Cell Bank of the Chinese Academy of Sciences, and the human normal prostate epithelial ATCC cell line was purchased from Wuhan Ponsure Life Science and Technology Co., Ltd.

### 2.2. Main Materials and Equipment

1,3-Diphenylisobenzofuran (DPBF) (Shanghai Aladdin Biochemical Technology Co., LTD.), calreticulin (CRT) antibody, high mobility group box 1 protein (HMGB1) antibody (Wuhan Sanying Biotechnology Co., LTD.), and 4% paraformaldehyde (Shanghai Yuli Biotechnology Co., LTD.) were used. An ultrasound machine (Ningbo Xinzhi Biotechnology Co., Ltd.), magnetic resonance imaging (MRI) equipment (3.0T, Philips from the Netherlands), an ultraviolet‒visible spectrophotometer (PerkinElmer from the United States), a particle size analyzer (Malvern Panalytical, UK), an inverted fluorescence microscope, a laser confocal microscope (Leica, Germany), a flow cytometer (Becton Dickinson, USA), and a scanning electron microscope (SEM) (Thermo Fisher Scientific, USA) were used.

### 2.3. Experimental Methods

#### 2.3.1. Preparation of Nanoprobes

This study adopted a prospective design, and the preparation method of drug self-assembly was used. Ten milligrams of Gem and 2 mg of Ce6 were dissolved in 1.5 mL of methanol, and an ultrasonic bath was used for 1 min to promote self-assembly of the drug. After the organic solvent was removed via rotary evaporation, 2 mL of ddH2O was added for dissolution, and the mixture was then ultrasonically treated for 1 minute to obtain uniformly dispersed GC nanoprobes. The samples were stored at 4°C in the dark for future use.

#### 2.3.2. Characterization of Nanoprobes

The morphology of the GC nanoprobes was observed via SEM, and their particle size, Polydispersity Index (PDI), and zeta potential were measured via a particle size analyzer. The peaks of Ce6 and Gem in the GC nanoprobes were detected via an ultraviolet spectrophotometer. The fluorescence spectra of Ce6 and GC before and after coincubation with acidic buffer solution at pH 5.0 were detected via a fluorescence spectrophotometer.

#### 2.3.3. Determination of Singlet Oxygen

The generation of singlet oxygen (1O_2_) by the GC nanoprobes under laser irradiation was detected by DPBF probes. DPBF was dissolved in the organic solvent DMSO (with a final concentration of 0.5 mg/mL). Thirty microlitres of DPBF solution was dissolved together with the GC nanoprobe solution containing 10 μmol/L Ce6 in 1 mL of ddH_2_O. After thorough mixing, the mixture was placed in a 96-well plate. The sample was subsequently irradiated with a laser at 660 nm (0.4 W/cm²) for 60 seconds. The absorption peaks at wavelengths ranging from 350 to 550 nm were detected via a UV-visible spectrophotometer.

#### 2.3.4. Drug Release at Different pH Values

The release capacity of the nanoprobes in an acidic microenvironment was verified via a dialysis drug release method. The pH 7.4 group and the pH 5.0 group were set up, with 3 parallel samples in each group. The GC nanoprobe mixture was added to the dialysis bags (with a cutoff molecular weight of 3,500 Da). The dialysis bags were placed in beakers containing solutions of pH 7.4 and pH 5.0 and were continuously stirred at a rotational speed of 200 r/min on a magnetic stirrer for 24 hours. The solution in 20 μL of the dialysis bag was drawn every hour and diluted 10 times. The ultraviolet characteristic absorption peak of the drug Gem at a wavelength of 269 nm was determined via ultraviolet‒visible light absorption spectroscopy. The formula for drug release was as follows: Gem/Ce6 cumulative release (%) = (D0 - Dt)/D0 × 100.

#### 2.3.5. Cytotoxicity Experiment

The toxic effects of the GC nanoprobes on the mouse prostate cancer PNEC30 cell line were detected via the MTT method. Five groups were set up in the experiment: The control group (PBS group), the Gem group, the GC nanoprobe group (GC group), the photosensitizer Ce6 combined with laser irradiation group (Ce6+L group), and the GC nanoprobe combined with laser irradiation group (GC+L group). PNEC30 cells were spread in 96-well plates and incubated. After the cells had adhered, culture media containing different concentrations of drugs (0.1, 1, 10, 50 and 100 μg/mL) were added, and the culture was continued for 4 hours. The Ce6+L group and the GC+L group were irradiated with a 660 nm laser (0.4 W/cm²) for 10 seconds and then incubated for 24 hours. Following the addition of 20 μL of MTT solution (5 mg/mL), the mixture was incubated for four hours. To make sure the crystals were completely dissolved, 150 μL of DMSO solution was added when the culture was stopped, and the mixture was gently agitated on a shaker for ten minutes. Using a microplate reader, the absorbance values of each well at a wavelength of 490 nm were calculated. The survival rate of the cells in each group was calculated according to the formula (D sample - D blank)/(D control - D blank) × 100%.

#### 2.3.6. Determination of ROS Generation at the Cellular Level

Mouse prostate cancer PNEC30 cells were evenly spread in 96-well plates. After the cells had adhered, different intervention regimens (PBS, 50 μg/mL Gem, GC, 10 μg/mL Ce6+L and GC+L) were used. One milliliter of DCFH-DA probe diluted with serum-free culture medium (1:1,000) was added to each well, and the mixture was incubated for 20 minutes in a cell incubator at 37°C. After the incubation was complete, the cells were washed with PBS, and the generation of intracellular ROS was detected via inverted fluorescence microscopy.

### 2.4. CEST Imaging Study of the GC Nanoprobes

Gem was prepared in solutions of different concentrations (0, 10, 20, and 30 mmol/L) with PBS for CEST imaging. The pH values of the GC nanoprobe solutions (with a Gem drug concentration of 20 mmol/L) were adjusted to 7.4 and 5.0. After incubation for 24 hours, CEST imaging was performed. 4D multisource transmission technology and mDIXON XD TSE acquisition technology were adopted. Imaging parameters: repetition time/echo time (TR/TE) = 5,864 ms/7.8 ms, field of view (FOV) = 230 mm×180 mm, voxel = 1.8 mm×1.8 mm matrix = 128×100, layer thickness = 1 mm, scan gap = 0 mm, number of layers = 10, average scan time = 1, saturation intensity (B1) = 2.0 μT, saturation time (tsat) = 2 s. The saturation RF pulse frequency of Gem is offset by the resonant frequency of water within the scanning range of -8 to 8 ppm (in conventional CEST scanning, the chemical shift of water is set to 0 ppm). Water-saturation shift referencing (WASSR) is used for the nonuniformity correction of field B0.

CEST image postprocessing process: Postprocessing analysis was carried out via MATLAB software, and the Z-spectrum (with the horizontal axis being the frequency offset (unit ppm) and the vertical axis being the intensity ratio of the saturated signal/unsaturated signal (S/S0)) and the magnetization transfer ratio asymmetry curves were plotted. (MTRasym) First, the CEST data are imported, and B0 field correction is carried out via the WASSR data. Then, postprocessing of the CEST signal is conducted through a series of processes, such as threshold denoising, interpolation, and quantitative analysis. The CEST signal is quantized by MTRasym = Ssat (-Δ ω)/S0- Ssat (Δ ω)/S0, where Ssat (-Δ ω), Ssat (Δ ω), and S0 represent the water signals with saturation frequency offsets of -Δ ω, Δ ω, and unsaturated (ω is the saturation pulse frequency), respectively.

### 2.5. Cell Uptake

Mouse prostate cancer PNEC30 cells in the logarithmic growth phase were uniformly distributed in 6-well plates at a density of 1×105 cells per well and incubated overnight. After the medium was removed, medium containing 50 μg/mL GC nanoprobes was added to each well, and the samples were incubated for different durations (0, 1, 2, 4, 6 and 12 h). After the incubation was complete, the cells were washed with PBS to remove residual nanoprobes. The fluorescence intensity of the cells was subsequently detected via flow cytometry and confocal microscopy to evaluate the ability of the PNEC30 cells to take up the GC nanoprobes.

### 2.6. Cell-Level Time-Dependent Activation of CEST Imaging

The GC nanoprobes were coincubated with mouse prostate cancer PNEC30 cells or normal prostate epithelium ATCC cells for 2 hours. The old culture medium was subsequently discarded, and new culture medium was added for continued culture for 0, 2, 4, 6 and 12 hours. The cells were collected and fixed in 1 mL of agarose (0.5%) for CEST scanning.

### 2.7. Detection of Pyroptosis Markers in Cells

After mouse prostate cancer PNEC30 cells were subjected to different protocols (PBS, Gem, GC, Ce6+L and GC+L), changes in cell morphology were observed under a bright-field microscope. The supernatants of PNEC30 cells treated with different drugs were collected and placed in 96-well plates. ELISAs were used to detect the concentrations of LDH, interleukin-1β (IL-1β) and IL-18 in the supernatants of PNEC30 cells after different treatments.

### 2.8. Immunofluorescence Detection

Mouse prostate cancer PNEC30 cells from different groups were collected. They were fixed with 4% paraformaldehyde for 10 minutes, washed with PBS, permeated with 0.5% Triton for 15 minutes, rinsed twice with PBS, blocked with 5% bovine serum albumin (BSA) for 30 minutes, and washed again with PBS. The samples were incubated overnight with CRT and HMGB1 antibodies (both diluted at 1:500) at 4°C. The next day, the sections were incubated with a goat anti-rabbit secondary antibody labeled with FITC in the dark for 1 - 2 hours and then rinsed 2 - 3 times with PBS, after which mounting agent containing DAPI was added. The fluorescence was detected via a fluorescence microscope.

### 2.9. In-vivo Imaging Study

A total of 1×106 mouse prostate cancer PNEC30 cells were subcutaneously injected into the right buttocks of each female BALB/c mouse to establish a subcutaneous tumor model in PNEC30 mice. GC nanoprobes and 50 mmol/L Gem were injected into the mice via the tail vein. Magnetic resonance scans were performed via a 3.0T MRI system at 0, 1, 4, 8 and 12 hours after injection. Imaging parameters: TR/TE = 5864 ms/7.8 ms, layer thickness = 1 mm, FOV = 230 mm×180 mm, matrix = 128 × 100, number of layers = 10, B1 = 2.0 µT, tsat = 2 s, saturation offset frequency from -5 to 5 ppm (water resonance frequency is set to 0 ppm). The total acquisition time was 8.5 minutes, and the equilibrium magnetization images (M0) collected were normalized. The T2-weighted imaging (T2WI) scanning parameters were as follows: TR/TE = 2,300 ms/60 ms, layer thickness = 1 mm, number of layers = 10, scanning gap = 0 mm, and FOV = 130 mm × 120 mm.

### 2.10. Evaluation of the Effects of PNEC30 on Pyroptosis and Tumor Suppression in Tumor-bearing Mice

A subcutaneous tumor model of prostate cancer in PNEC30 mice was constructed. The tumor-bearing mice were randomly assigned to one of five groups (n = 6) once the tumor volume had grown to around 100 mm: the control group, Gem group, GC group, Ce6+L group, and GC+L group. The mice received injections of all formulations via the tail vein. The Ce6+L and GC+L groups were subjected to laser irradiation for 5 minutes 4 hours after drug injection. The mice in each group were treated once every 2 days for a total of 4 treatments. During this period, the changes in the tumor volume of the mice were recorded (tumor volume = long diameter × wide diameter 2/2). On the 14th day of treatment, the serum of the mice was collected, and the concentrations of IL-1β and IL-18 were detected via ELISA kits. The mice were subsequently euthanized, and the tumor tissues were collected for H&E staining; Ki67 and cleaved caspase-3 immunohistochemical staining; and HMGB1 and CRT immunofluorescence staining. The tumor tissues were analyzed, and the effects of pyroptosis in each treatment group were compared.

### 2.11. Observation of the Histological Morphology of the Main Organs in Mice After Treatment

After the antitumor treatment was completed, the mice were euthanized, organs such as the heart, liver, spleen, lungs and kidneys were collected, and the tissues were fixed with 4% paraformaldehyde. The sample tissues were subsequently dehydrated with gradient ethanol, treated with a transparent agent, immersed in wax, and embedded to fix the shape, after which the wax blocks were sectioned. The paraffin in the sections was removed from the slides using xylene and ethanol. After H&E staining, the tissue morphology was observed under a microscope.

### 2.12. Diagnostic Accuracy Index Method

Using pathological examination as the gold standard, the diagnostic accuracy of CEST imaging was evaluated through a systematic approach. First, we conducted histopathological analysis on the mouse prostate cancer model to determine the exact location and boundaries of the tumors and matched them with the CEST signals. Based on this, we calculated the sensitivity (true positive rate) and specificity (true negative rate) of CEST imaging for detecting tumors. Sensitivity was defined as the proportion of tumor samples with positive CEST signals among all pathologically confirmed tumor samples, and specificity was defined as the proportion of normal tissue samples with negative CEST signals among all pathologically confirmed normal tissue samples. At the same time, we drew the receiver operating characteristic (ROC) curve and evaluated the overall diagnostic efficacy of CEST imaging by calculating the area under the curve (AUC). Additionally, we calculated the positive predictive value and negative predictive value to assess the practical value of CEST imaging in clinical applications. To compare the differences in diagnostic accuracy between different time points and different treatment groups, we used the McNemar test for paired analysis and calculated the Kappa coefficient to evaluate the consistency between CEST imaging and pathological results. All diagnostic accuracy indicators were statistically analyzed using 95% confidence intervals to ensure the scientific and reliable nature of the results.

### 2.13. Statistical Analysis

The data were statistically analyzed via GraphPad Prism 9.1.1 software. The quantitative data are expressed as the means ± s. Analysis of the differences between the two data groups was performed using an independent-samples t test. Multiple groups were compared for differences using a one-way ANOVA. If the differences were statistically significant, the LSD test was used for comparisons between groups. P < 0.05 indicated a statistically significant difference.

## 3. Results

### 3.1. Characterization of the GC Nanoprobes

**Figure 1.**
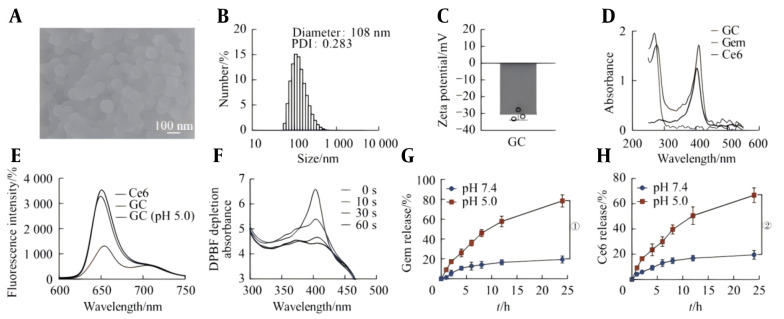
Characterization of the GC nanoprobes and their acid-responsive drug release capabilities. A, scanning electron microscope (SEM) image of the GC nanoprobes; B, hydrodynamic size distribution of the GC nanoprobes; C, zeta potential of the GC nanoprobes; D, UV‒visible absorption spectra of gemcitabine (Gem), Ce6, and GC nanoprobes; E, fluorescence spectra of the GC nanoprobes and Ce6; F, UV‒visible absorption spectra of DPBF at different time points after laser irradiation of the water solution of the GC nanoprobes; G/H, release of Gem (G) and Ce6 (H) at pH 5.0 and pH 7.4.

SEM revealed that the self-assembled GC nanoprobes had good dispersibility and presented a quasi-spherical structure, with a particle size of approximately 108 nm, a PDI of approximately 0.283, and a zeta potential of 31.0 ± 2.7 mV (Figure 1A–C). The ultraviolet-visible light absorption spectra revealed that the GC nanoprobe had strong absorption peaks at wavelengths of 270 nm and 410 nm and was somewhat offset compared with the characteristic absorption peaks of free Ce6 (402 nm) and Gem (269 nm) (Figure 1D). Furthermore, the fluorescence spectra revealed that, compared with free Ce6, the self-assembled GC nanoprobes exhibited fluorescence quenching. However, the GC fluorescence signal was activated at pH 5.0 (Figure 1E). The above results indicate the successful preparation of the GC nanoprobes.

### 3.2. 1O2 generation Capacity of GC and Its Drug Release Capacity in An Acidic Microenvironment

#### 3.2.1. Generation of 1O2

DPBF can combine with 1O_2_ and be irreversibly oxidized. Therefore, the efficiency of 1O2 generation can be evaluated by measuring the consumption of DPBF (Figure 1F). After laser irradiation treatment of the GC nanoprobe solution, ultraviolet‒visible light absorption spectroscopy revealed that the absorption peak of DPBF at a wavelength of 410 nm was significantly reduced in a time-dependent manner, indicating that the GC nanoprobe can efficiently generate 1O2 during the PDT process. This is helpful for inducing pyroptosis in tumor cells in the later stage, thereby activating the antitumor immune response.

#### 3.2.2. Drug Release

The formation of GC nanoprobes relies on π-π stacking and hydrophobic interactions between the drug Gem and the photosensitizer Ce6 molecules, which makes the nanoprobes sensitive to pH. To verify the drug release capacity of the drug-loaded nanoprobe in an acidic microenvironment, the GC nanoprobes were placed in release media with different pH values to calculate the cumulative release amounts of Gem and Ce6 (Figure 1G-H). The drug release rate of the nanoprobe in the buffer solution at pH 7.4 was slow, and the cumulative drug release within 24 hours did not exceed 20%. This might be due to the continuous oscillation of the nanoprobe at 37°C, which led to a decrease in stability and a small amount of drug release. Under acidic conditions at pH 5.0, the cumulative amounts of Gem and Ce6 released within 24 h reached 80% and 65%, respectively. GC nanoprobes can be effectively decomposed in the acidic microenvironment of tumors.

### 3.3. Cytotoxicity and Intracellular Reactive Oxygen Species Generation

**Figure 2.**
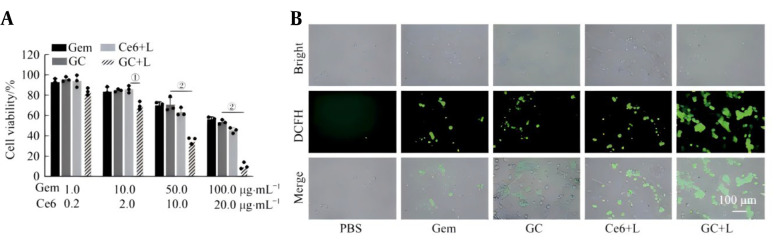
The effects of GC nanoprobes combined with laser irradiation on the proliferation and intracellular reactive oxygen species (ROS) generation of mouse prostate cancer PNEC30 cells. A, effects of different concentrations of GC nanoprobes combined with laser irradiation (0.4 W/cm^2^) on the proliferation of PNEC30 cells; B, ROS detection in PNEC30 cells after different treatments

After treating mouse prostate cancer PNEC30 cells with different concentrations of Gem nanoprobes, the activity of the PNEC30 cells was detected via the MTT method. The results showed that the killing effect of the GC nanoprobes on tumor cells was concentration-dependent: when the GC concentration was 50 μg/mL, the nanoprobes could effectively kill tumor cells. However, under combined treatment with laser irradiation, the cell survival rate of the experimental group decreased significantly (P < 0.05), suggesting that the GC nanoprobe has good tumor suppression ability and can achieve photodynamic sensitization of pyroptosis (Figure 2A). Furthermore, the production of ROS in PNEC30 cells was evaluated via the use of the probe DCFH-DA. The results revealed a large amount of green fluorescence in the GC combined with laser irradiation group, indicating that a large amount of cytotoxic ROS was produced in PNEC30 cells sensitized by PDT (Figure 2B). 

### 3.4. In-vitro CEST Activation Imaging

**Figure 3.**
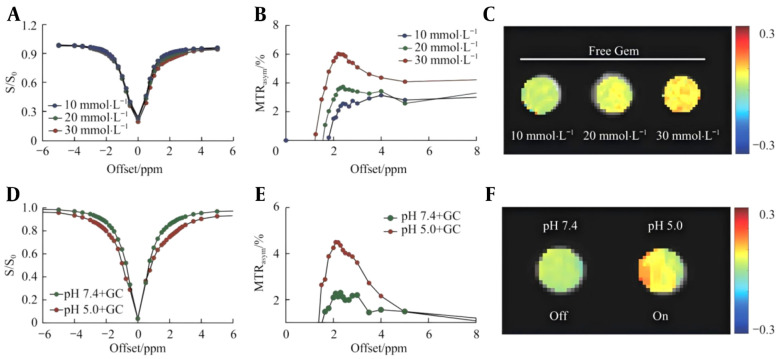
In-vitro chemical exchange saturation transfer (CEST) imaging of Gem at different concentrations and of GC nanoprobes at different pH values

Chemical exchange saturation transfer imaging of Gem at different concentrations revealed that the CEST signal increased in a concentration-dependent manner. CEST characteristic peaks can be observed in the Z-spectrum and MTRasym near a frequency offset of approximately 2 ppm (Figure 3A to C), which is consistent with previous research results. CEST imaging scans were performed on the GC nanoprobes under different pH conditions. The Z-spectra and MTRasym data analyzed by MATLAB software are shown in Figure 3D and E. Compared with the same concentration of free Gem (20 mmol/L), obvious signal quenching (Off) was observed in the GC nanoprobe CEST image at pH 7.4, whereas in the acidic microenvironment at pH 5.0, the CEST signal was significantly activated (On) (Figure 3F). The above analysis indicates that the CEST signal of the GC nanoprobes is significantly enhanced under acidic conditions at pH 5.0, suggesting that the GC nanoprobes lyse in an acidic microenvironment. The release of Gem leads to the exposure of imaging groups, thereby specifically activating the CEST signal.

### 3.5. Uptake of GCs by Prostate Cancer Cells in Mice

**Figure 4.**
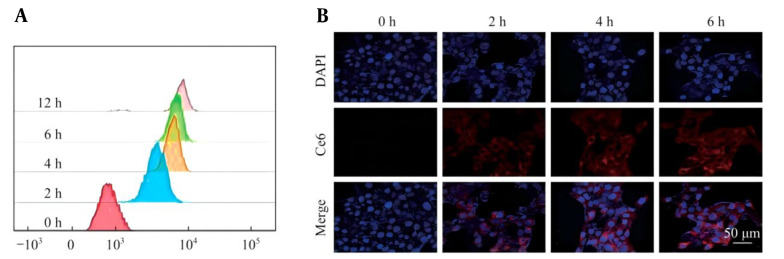
Uptake of GC nanoprobes in PNEC30 cells of mouse prostate cancer

Effective endocytosis is the key to drug accumulation for the treatment of tumors. The uptake of GC nanoprobes by PNEC30 cells in mouse prostate cancer was determined by flow cytometry. The results showed that the GC nanoprobes could be effectively taken up by PNEC30 cells and reached the maximum cumulative amount within 4 to 6 hours (Figure 4A). In addition, the results of laser confocal fluorescence imaging revealed the same outcome (Figure 4B). Therefore, for the subsequent experiments, laser irradiation was chosen to treat the cells 4 hours after the addition of the GC probe or drug.

### 3.6. Cell-Level Time-Dependent Chemical Exchange Saturation Transfer Activation Imaging

**Figure 5.**
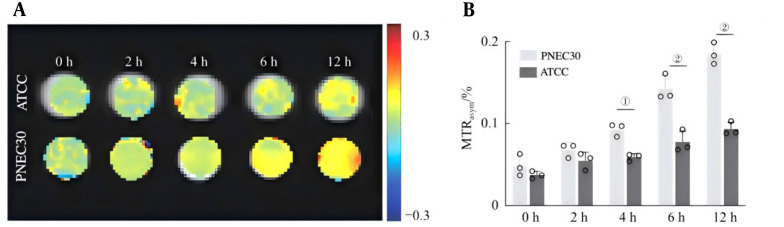
Responsive chemical exchange saturation transfer (CEST) activation imaging of GC nanoprobes in tumor cells and normal cells

To verify the pH response and time-dependent CEST activation imaging of the GC nanoprobes at the cellular level, mouse prostate cancer PNEC30 cells and normal prostate epithelial ATCC cells were coincubated with the GC nanoprobes. The activation effects of CEST at different time points (0, 2, 4, 6 and 12 hours) were subsequently monitored. As shown in Figure 5, after PNEC30 cells in an acidic tumor microenvironment were incubated with GC nanoprobes, the CEST signal and the quantitative value of MTRasym significantly increased and increased with increasing incubation time. However, no obvious change was observed in the CEST signal in normal prostate epithelial ATCC cells. The differences between the two groups at each time point from 4 - 12 hours were statistically significant (all P < 0.05). This result indicates that the acidic microenvironment of prostate cancer cells can cause GC nanoprobes to lyse, releasing Gem and exposing the amide groups on its surface to achieve CEST activation imaging.

### 3.7. Pyroptosis-Mediated Immune Response

**Figure 6.**
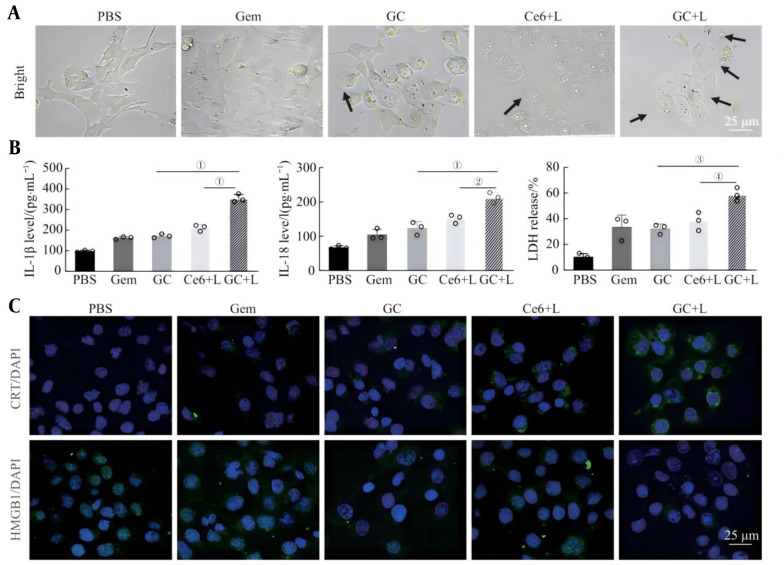
Induction of the pyroptosis ability of PNEC30 cells and triggering of the immune response by GC nanoprobes under PDT sensitization

The microscopic observation results (Figure 6A) revealed that a small number of balloon-like bubbles began to appear in mouse prostate cancer PNEC30 cells pretreated with the GC nanoprobes, suggesting the occurrence of pyroptosis. After laser irradiation, obvious cell swelling and exudation of the cell contents were observed in the GC+L group. To further verify the above results, the levels of LDH, IL-1β and IL-18 in the cell supernatants of the different treatment groups were detected. The results revealed that the levels of LDH and inflammatory cytokines in the experimental group were significantly increased, indicating that PNEC30 cells underwent pyroptosis. The integrity of the cell membrane was disrupted, leading to the release of intracellular inflammatory contents (Figure 6B). Pyroptosis can induce a strong immune response in a short time and effectively inhibit tumor growth by triggering ICD. During the occurrence of ICD, DAMPs are released, including the eversion of CRT on the cell membrane surface and the release of HMGB1 in the nucleus. The results of CRT immunofluorescence staining revealed that, compared with that in the PBS group, the green fluorescence in the Gem, GC and Ce6+L groups increased to varying degrees. More significant CRT fluorescence was observed in the cells of the GC+L group than in those of the other groups, indicating that a large amount of CRT was expressed on the cell membrane surface. The results of HMGB1 immunofluorescence staining revealed that the intracellular green fluorescence in the GC+L group was significantly weakened, indicating that HMGB1 was effectively released extracellularly (Figure 6C). The above research results indicate that GC nanodrug-loaded probes can effectively induce ICD and initiate adaptive immune responses through photosensitization via pyroptosis.

### 3.8. In-vivo Chemical Exchange Saturation Transfer Activation Imaging Study

The tail vein of tumor-bearing mice was injected with GC nanoprobes or 50 mmol/L Gem, and CEST imaging was performed at different time points. The results of the analysis of the CEST images and MTRasym values are shown in Figure A 7 and B. As time progressed, the CEST signal gradually increased and reached its peak at 4 hours, after which the signal gradually decreased. Free Gem was injected as a control. The results revealed that the CEST signal rapidly increased and then rapidly decreased within 1 hour. The above results suggest that, owing to the EPR effect of the GC nanoprobes, the CEST signal at the tumor site is enhanced, and its imaging effect is superior to that of free Gem, indicating that this nanoprobe can be used as a potential contrast agent for in vivo CEST imaging.

**Figure 7.**
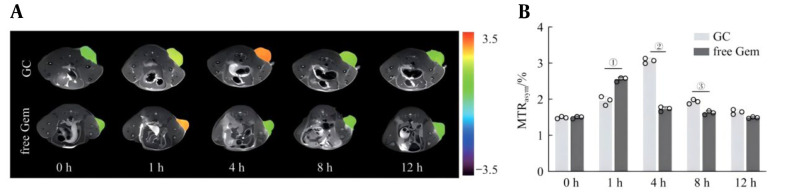
I-vivo chemical exchange saturation transfer (CEST) imaging of the GC nanoprobes

### 3.9. In-vivo Antitumor Effects Mediated by Pyroptosis

**Figure 8.**
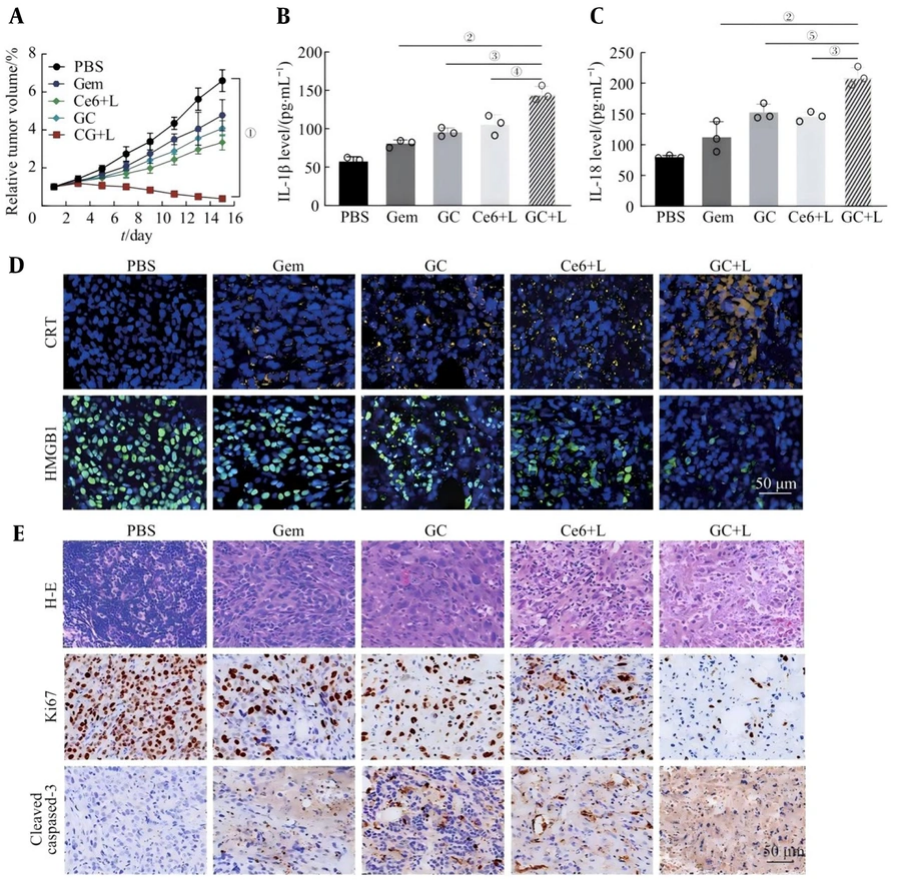
In-vivo antitumor effect triggered by pyroptosis in GC nanoprobes

To evaluate the in vivo antitumor effects of the GC nanoprobes, PBS, Gem, or GC nanoprobes were injected into each group of mice through the tail vein. The Ce6+L group and the GC+L group received laser irradiation at the tumor sites 4 hours after drug injection. Changes in the tumor volume of the mice in each group were observed within 14 days. As shown in Figure 8A, the weights of the mice in the PBS group and the Gem group were relatively.

The tumor volume increased rapidly. In contrast, the relative tumor volume growth of mice in the Ce6+L group and the GC group was relatively slow during the treatment process. The GC nanoprobes under photodynamic synergy therapy demonstrated significant tumor suppression capabilities. After three treatments, the tumor volume gradually decreased, and in the later stage, it was completely ablated. In addition, through the detection of the serum of the mice in each group, the levels of inflammatory factors such as IL-1β and IL-18 in each group of mice increased to varying degrees. Among them, the levels of inflammatory factors in the GC+L group were significantly greater than those in the PBS group, indicating that tumor pyroptosis under photodynamic synergy led to an increase in inflammatory factors in the mice (Figure 8B and C). Immunofluorescence staining of tumor tissues revealed that in the GC+L group, the tumor cells had high levels of CRT release and HMGB1 release, confirming that the photosensitized GC probe triggered the immune response of the body by inducing pyroptosis (Figure 8D). In addition, the results of H&E staining of tumor tissues and immunohistochemical staining of Ki67 and cleaved caspase-3 confirmed that, compared with the other treatment groups, the GC+L group had a greater ability to resist tumor cell proliferation and induce tumor cell apoptosis. The above experimental results indicate that pyroptosis mediated by GC nanoprobes under photosensitization can exert powerful antitumor effects by triggering an immune response in vivo (Figure 8E). 

## 4. Discussion

The various inflammatory mediators released during pyroptosis can induce a powerful immune response in the body, indicating great potential in the treatment of prostate cancer (16-18). In recent years, various metal ions and small-molecule drugs have been designed for the immunotherapy of prostate cancer via pyroptosis. However, pyroptosis caused by some cytotoxic drugs in an uncontrollable or nontargeted manner can damage normal tissues of the body, often raising biosafety issues. The nanodrug delivery system can improve pharmacokinetics, achieve targeted tumor delivery, promote the release of targeted drugs in response to the tumor microenvironment, and has important application value in regulating pyroptosis in tumors (19). However, single-response stimulation can easily lead to low drug release efficiency and poor therapeutic effects. To further enhance the efficiency of tumor treatment based on pyroptosis, this study adopted a dual regulatory strategy involving the endogenous acidic tumor microenvironment response and exogenous laser irradiation (20-22). Through the drug self-assembly method, responsive nanoprobe GCs were successfully prepared to achieve precise drug release and significantly enhance the antitumor immune response by activating the pyroptosis pathway. This probe lyses and releases the antitumor drug Gem in an acidic microenvironment to induce pyroptosis in prostate cancer cells. In addition, under PDT sensitization, a large amount of cytotoxic ROS can be generated, further amplifying the pyroptosis effect at the tumor site. The detection of the in vivo and in vitro pyroptosis-related markers IL-1β and IL-18, as well as the ICD markers CRT and HMGB1, confirmed that GC nanoprobes can induce pyroptosis in prostate cancer cells under the synergistic effects of PDT, triggering an immune response to achieve improved tumor suppression (23).

It is highly important to visualize the pyroptosis process of prostate cancer in real time by using a new imaging strategy during the treatment of prostate cancer (24-26). Traditional imaging techniques such as conventional MRI have limitations such as low sensitivity and susceptibility to interference from metabolites in the body, and visualizing the progress of tumor treatment at the molecular level is often difficult (27). However, CEST imaging has advantages such as high sensitivity and noninvasiveness and has research value and application potential in the precise and noninvasive visualization of the treatment process and therapeutic effect evaluation of prostate tumors. The GC nanoprobes prepared in this study demonstrated excellent effects in promoting pyroptosis in tumors (28-30). In addition, CEST signal-specific activation can be achieved in the acidic microenvironment of tumors by loading Gem with amide CEST imaging protons (31). This nanoprobe does not need to be labeled and can be highly sensitively detected by CEST through the exchange of hydroxylamide and hydroxyl protons with water molecules (32). Therefore, nanoprobes based on Gem-CEST imaging can be used for real-time noninvasive visualization of the pyroptosis process in prostate cancer (33-35). The CEST imaging signal of the GC nanoprobe is quenched and presents an "off" state at pH 7.4, whereas in the simulated acidic microenvironment, it shows a specific CEST activation imaging effect, "on". This shift from "off" to "on" may be attributed to the disruption of the structural stability of the spherical nanoprobes in an acidic microenvironment, which leads to the full exposure of exchangeable protons such as amides in the drug, thereby enhancing the CEST signal. The GC nanoprobes have a specific CEST activation effect on the tumor microenvironment. Specifically, the CEST signal at the tumor site gradually increased over time and reached its peak at 4 h, indicating that it has a good in vivo imaging effect and can be used as an in vivo CEST contrast agent to guide pyroptosis tumor treatment and efficacy evaluation.

### 4.1. Conclusions

In summary, this study successfully constructed GC nanoprobes, characterized their morphology, particle size, etc., and verified their excellent photodynamic sensitization pyroptosis and CEST activation effects both in vivo and in vitro. The "off-one effect" of this nanoprobe in the acidic microenvironment of tumors can be utilized to visualize the drug release process noninvitatively and evaluate the therapeutic effect on the basis of pyroptosis. In the future, a correlation analysis between pyroptose-related biomarkers and changes in CEST signals can dynamically reflect the expression levels of pyroptose-related molecules in vivo. It is expected to achieve noninvasive visualization of the diagnosis and treatment process of prostate cancer on the basis of pyroptosis at the molecular level, which is highly important for precise tumor treatment guided by imaging technology.

## Data Availability

The dataset presented in the study is available on request from the corresponding author during submission or after publication.

## References

[1] Wahnou H, Youlyouz-Marfak I, Liagre B, Sol V, Oudghiri M, Duval RE (2023). Shining a Light on Prostate Cancer: Photodynamic Therapy and Combination Approaches.. Pharmaceutics..

[2] Omiyale OC, Musa M, Otuyalo AI, Gbayisomore TJ, Onikeku DZ, George SD (2023). A review on selenium and gold nanoparticles combined photodynamic and photothermal prostate cancer tumors ablation.. Discov Nano..

[3] Derks YHW, Schilham MGM, Rijpkema M, Smeets EMM, Amatdjais-Groenen HIV, Kip A (2023). Imaging and photodynamic therapy of prostate cancer using a theranostic PSMA-targeting ligand.. Eur J Nucl Med Mol Imaging..

[4] Uddin MMN, Bekmukhametova A, Antony A, Barman SK, Houang J, Wu MJ (2023). Photodynamic Treatment of Human Breast and Prostate Cancer Cells Using Rose Bengal-Encapsulated Nanoparticles.. Molecules..

[5] Yang J, Wang Y, Yu W, Guan Y, Amponsah PEO, Tang X (2024). Comparative Evaluation of Antitumor Chinese Patent Medicine Injections Based on Evidence-Based Medicine and Markov Model.. World J Tradit Chin Med..

[6] Luo D, Wang X, Ramamurthy G, Walker E, Zhang L, Shirke A (2023). Evaluation of a photodynamic therapy agent using a canine prostate cancer model.. Prostate..

[7] Atmaca GY, Aksel M, Bilgin MD, Erdogmus A (2023). Comparison of sonodynamic, photodynamic and sonophotodynamic therapy activity of fluorinated pyridine substituted silicon phthalocyanines on PC3 prostate cancer cell line.. Photodiagnosis Photodyn Ther..

[8] Saldutto P, Cavacece F, Rocca R, Di Mauro E, Verratti V, Sangiorgi GM (2025). The Safety and Efficacy of Vascular-Targeted Photodynamic Therapy in Low-Risk Prostate Cancer.. Cancers (Basel)..

[9] Hochma E, Ishai PB, Firer MA, Minnes R (2024). Phyto-Photodynamic Therapy of Prostate Cancer Cells Mediated by Yemenite 'Etrog' Leave Extracts.. Nutrients..

[10] Xu Y, Tan Q, Sun C, Jia Y, Li S, Yang X (2025). Photodynamic therapy for the precise treatment of localized prostate cancer.. Front Oncol..

[11] Wu H, Dai W, Wang L, Zhang J, Wang C (2023). Comprehensive Analysis of the Molecular Mechanism for Gastric Cancer Based on Competitive Endogenous RNA Network.. World J Tradit Chin Med..

[12] Kim Y, Mondal S, Shin H, Tak S, Doan VHM, Oh J (2025). Advanced Precision Dual Photothermal and Photodynamic Therapy for Prostate Cancer Using PSMA-ICG-Conjugated Gold Nanorods.. ACS Biomater Sci Eng..

[13] Hochma E, Firer MA, Minnes R (2025). Near-Infrared and Sono-Enhanced Photodynamic Therapy of Prostate Cancer Cells Using Phyto-Second Harmonic Generation Nanoconjugates.. Polymers (Basel)..

[14] Solyanik O, Chaloupka M, Clevert DA, Schmidt VF, Ingenerf M, Kazmierczak P (2024). Prospective close monitoring of the effect of vascular-targeted photodynamic therapy and high intensity focused ultrasound of localized prostate cancer by multiparametric magnetic resonance imaging.. World J Urol..

[15] Capozza M, Digilio G, Gagliardi M, Tei L, Marchesi S, Terreno E (2024). Silicon Phthalocyanines Functionalized with Axial Substituents Targeting PSMA: Synthesis and Preliminary Assessment of Their Potential for PhotoDynamic Therapy of Prostate Cancer.. ChemMedChem..

[16] Hana H, Qian C, Song M, Zhang T, Yang C, Gu R (2023). The Mechanism of Total Ginseng Extracts in the Treatment of Lung Cancer Progression Based on Network Pharmacology and Experimental Validation.. World J Tradit Chin Med..

[17] Gao L, Tang Z, Xiao D, Chen X, Zhu Y (2025). Prostate Cancer-Targeting Liposome Loaded with Zinc Ion-Coordinated Photosensitizer for Enhanced Chemo-Photodynamic Therapy.. Pharmaceutics..

[18] Azad AK, Dinakaran D, Moore RB (2025). Radiation activated photodynamic therapy (radioPDT) induces lipid peroxidation and vascular mediated tumor regression of prostate cancer.. Sci Rep..

[19] Serag E, Abdel Gaber SA, Abdel-Shafi AA, El-Khouly ME (2025). Perylene tetracarboxylic acid-folate conjugated carbon quantum dots for targeted photodynamic therapy of prostate cancer.. Bioorg Chem..

[20] Bakay E, Pamukcu A, Sen Karaman D, Topaloglu N (2025). Apoptosis-inducing photodynamic therapy via dual-wavelength responsive ICG/Ce6 incorporated mesoporous silica nanoparticles in prostate cancer cells.. Photochem Photobiol Sci..

[21] Czaja A, Datta M, Rak J, Zdrowowicz M (2026). Halogen substituted 4-thio-2'-deoxyuridines as photosensitizers for the photodynamic therapy of prostate cancer. An in vitro study.. Spectrochim Acta A Mol Biomol Spectrosc..

[22] Li D, Hou W, Hua B, Zhang P, Xiong L, Liu H (2023). Shenyi Capsule Prolongs Postoperative Survival of Patients with Nonsmall Cell Lung Cancer.. World J Tradit Chin Med..

[23] You Y, Liang X, Yin T, Chen M, Qiu C, Gao C (2018). Porphyrin-grafted Lipid Microbubbles for the Enhanced Efficacy of Photodynamic Therapy in Prostate Cancer through Ultrasound-controlled In Situ Accumulation.. Theranostics..

[24] Thakur PS, Pramanik SD, Roy P, Sankar M (2025). Development of 3D Porphyrinic Covalent Organic Frameworks for Enhanced Sono-Photodynamic Therapy against Prostate Cancer.. Inorg Chem..

[25] Zhang XW, Li L, Liao M, Liu D, Rehman A, Liu Y (2024). Thermal Proteome Profiling Strategy Identifies CNPY3 as a Cellular Target of Gambogic Acid for Inducing Prostate Cancer Pyroptosis.. J Med Chem..

[26] Zhao Q, Zuo KW, Li WP (2024). [Pyroptosis in the development and progression of prostate cancer: Progress in research].. Zhonghua Nan Ke Xue..

[27] Zhu M, Liu D, Liu G, Zhang M, Pan F (2023). Caspase-Linked Programmed Cell Death in Prostate Cancer: From Apoptosis, Necroptosis, and Pyroptosis to PANoptosis.. Biomolecules..

[28] Liu Z, Kuang S, Chen Q (2023). A review focusing on the role of pyroptosis in prostate cancer.. Medicine (Baltimore)..

[29] Wu F, Wang M, Zhong T, Xiao C, Chen X, Huang Y (2023). Inhibition of CDC20 potentiates anti-tumor immunity through facilitating GSDME-mediated pyroptosis in prostate cancer.. Exp Hematol Oncol..

[30] Wang H, He Z, Gao Y, Feng D, Wei X, Huang Y (2023). Dual-Pronged Attack: pH-Driven Membrane-Anchored NIR Dual-Type Nano-Photosensitizer Excites Immunogenic Pyroptosis and Sequester Immune Checkpoint for Enhanced Prostate Cancer Photo-Immunotherapy.. Adv Sci (Weinh)..

[31] Wolf I, Storz J, Schultze-Seemann S, Esser PR, Martin SF, Lauw S (2024). A new silicon phthalocyanine dye induces pyroptosis in prostate cancer cells during photoimmunotherapy.. Bioact Mater..

[32] Fu S, Qin S, Rausch W (2023). Chinese Herb Formulae Inhibit the Proliferation of Human Colon Cancer SW480 Cells by Inducing Cell Apoptosis.. World J Tradit Chin Med..

[33] Chen X, Kadier M, Shi M, Li K, Chen H, Xia Y (2025). Targeting Melatonin to Mitochondria Mitigates Castration-Resistant Prostate Cancer by Inducing Pyroptosis.. Small..

[34] Zeng Y, Li MX, Wu SQ, Xu C (2024). Carvedilol induces pyroptosis through NLRP3-caspase1-ASC inflammasome by nuclear migration of NF-kappaB in prostate cancer models.. Mol Biol Rep..

[35] Tilki D, van den Bergh RCN, Briers E, Van den Broeck T, Brunckhorst O, Darraugh J (2024). EAU-EANM-ESTRO-ESUR-ISUP-SIOG Guidelines on Prostate Cancer. Part II-2024 Update: Treatment of Relapsing and Metastatic Prostate Cancer.. Eur Urol..

